# Silencing of PARP2 Blocks Autophagic Degradation

**DOI:** 10.3390/cells9020380

**Published:** 2020-02-07

**Authors:** Laura Jankó, Zsanett Sári, Tünde Kovács, Gréta Kis, Magdolna Szántó, Miklós Antal, Gábor Juhász, Péter Bai

**Affiliations:** 1 Department of Medical Chemistry, Faculty of Medicine, University of Debrecen, H-4032 Debrecen, Hungary; janko.laura@med.unideb.hu (L.J.); sari.zsanett@med.unideb.hu (Z.S.); kovacs.tunde@med.unideb.hu (T.K.); mszanto@med.unideb.hu (M.S.); 2 Department of Anatomy, Histology and Embryology, Faculty of Medicine, University of Debrecen, H-4032 Debrecen, Hungary; greta@anat.med.unideb.hu (G.K.); antal@anat.med.unideb.hu (M.A.); 3 Institute of Genetics, Biological Research Centre, H-6726 Szeged, Hungary; gabor.juhasz@ttk.elte.hu; 4 Department of Anatomy, Cell and Developmental Biology, Eötvös Loránd University, H-1117 Budapest, Hungary; 5 MTA-DE Lendület Laboratory of Cellular Metabolism, H-4032 Debrecen, Hungary; 6 Research Center for Molecular Medicine, Faculty of Medicine, University of Debrecen, H-4032 Debrecen, Hungary

**Keywords:** PARP2, ARTD2, autophagy, LC3, AMPK, mTOR, PARP, nicotinamide-riboside, SIRT1

## Abstract

Poly(ADP-Ribose) polymerases (PARPs) are enzymes that metabolize NAD^+^. PARP1 and PARP10 were previously implicated in the regulation of autophagy. Here we showed that cytosolic electron-dense particles appear in the cytoplasm of C2C12 myoblasts in which PARP2 is silenced by shRNA. The cytosolic electron-dense bodies resemble autophagic vesicles and, in line with that, we observed an increased number of LC3-positive and Lysotracker-stained vesicles. Silencing of PARP2 did not influence the maximal number of LC3-positive vesicles seen upon chloroquine treatment or serum starvation, suggesting that the absence of PARP2 inhibits autophagic breakdown. Silencing of PARP2 inhibited the activity of AMP-activated kinase (AMPK) and the mammalian target of rapamycin complex 2 (mTORC2). Treatment of PARP2-silenced C2C12 cells with AICAR, an AMPK activator, nicotinamide-riboside (an NAD^+^ precursor), or EX-527 (a SIRT1 inhibitor) decreased the number of LC3-positive vesicles cells to similar levels as in control (scPARP2) cells, suggesting that these pathways inhibit autophagic flux upon PARP2 silencing. We observed a similar increase in the number of LC3 vesicles in primary PARP2 knockout murine embryonic fibroblasts. We provided evidence that the enzymatic activity of PARP2 is important in regulating autophagy. Finally, we showed that the silencing of PARP2 induces myoblast differentiation. Taken together, PARP2 is a positive regulator of autophagic breakdown in mammalian transformed cells and its absence blocks the progression of autophagy.

## 1. Introduction

Poly(ADP-ribose) (PAR) metabolism is an evolutionarily conserved posttranslational modification of proteins [1]. PAR is a branched polymer of ADP-ribose derived from NAD^+^ by its enzymatic cleavage. PAR is synthesized by members of the Poly(ADP-Ribose) polymerase enzyme family, among them, PARP1 and PARP2 [2]. PARP2 can be activated by binding to DNA or RNA through its *N*-terminus or through signaling pathways [2,3,4]. When activated, PARP2 accounts for 15–20% of total cellular PARP activity [5,6]. Both enzymes are involved in a plethora of cellular processes, among these, in the regulation of cellular energy and metabolic homeostasis [1,7,8,9]. 

Autophagy is a process in cells that is dedicated to the removal of damaged cellular proteins and components (e.g., mitochondria) [10,11]. The process is well-conserved across evolution. Damaged cellular components are engulfed by a biomembrane that is pickled with LC3 protein that is an accepted biomarker of autophagy [12]. The resulting autophagosomes then fuse with and are degraded in acidic lysosomes, a process that can be inhibited by chloroquine, an acidification inhibitor that prolongs the half-life of LC3-positive vesicles [12]. Autophagic flux is tightly linked to the bioenergetic output of cells through energy sensors, such as AMP-activated kinase (AMPK), mechanistic target of rapamycin (mTOR), PARPs, and SIRT1 [13,14,15]. The dysregulation of autophagy has a pathogenic role in disorders, such as cancer or metabolic diseases [10,11].

PARP10 [16] and PARP1 [17] were shown to be involved in the regulation of autophagy. PARP1 may act as a pro-autophagy factor [17,18,19,20,21,22,23,24,25], where its activation promotes autophagy under numerous conditions that are associated with DNA damage (DNA intercalators, irradiation, oxidative stress, heavy metal intoxication, ultraviolet B (UVB) radiation, etc.) [17,18,19,26,27,28,29,30]. In line with that, genetic silencing or pharmacological inhibition of PARP1 suppressed autophagy [17,18,19]. In our current study we showed an opposite impact of PARP2 on autophagy as compared to PARP1.

## 2. Material and Methods

### 2.1. Chemicals

All chemicals were from Sigma-Aldrich (St. Louis, MO, USA) unless stated otherwise. The source of key chemicals are listed in Table 1.

### 2.2. Cell Culture

PARP2-silenced C2C12 cells were described in [31]. C2C12 cells are of murine origin. C2C12 cells were maintained in DMEM (Sigma-Aldrich, 4500 mg/L glucose) containing 10% fetal bovine serum (FBS), 1% penicillin/streptomycin, and 2 mM l-glutamine at 37 °C with 5% CO_2_. In C2C12 cells, PARP2 was silenced by specific shRNA (the sequence was tested in [32]) that was maintained over extended periods (the cell line was created in 2010) by selection under 2.5 µg/mL for C2C12 cells [31]. Cells containing the control, non-specific sequence are termed scPARP2, while those containing the PARP2-specific shRNA are termed shPARP2 cells. Cells were differentiated in DMEM (Sigma-Aldrich, 1000 mg/L glucose) containing 2% horse serum for 4 days.

Male primary murine embryonic fibroblasts (MEFs) were created in the frame of a previous study [32]. Cells were maintained in DMEM (Sigma-Aldrich, 4500 mg/L glucose) containing 20% FBS, 1% penicillin/streptomycin, and 2 mM l-glutamine at 37 °C with 5% CO_2_.

### 2.3. Transient Transfection

Silencer select siRNAs were obtained from Thermo Fisher Scientific (Walthman, MA, USA). siRNAs targeting PARP2 (cat. no. 4390771, ID: s62056 as #1, s62057 as #2), SIRT1 (cat. no. 4390771, ID: s96766), and negative control (cat. no. 4390843) were used. Cells were seeded into a 24-well plate and transfected with siRNA at a final concentration of 30 nM using Lipofectamine RNAiMax (Invitrogen, Carlsbad, CA, USA) transfection reagent. Cells were incubated for 48 h.

### 2.4. Immunofluorescence and Confocal Microscopy

Cells were seeded on glass coverslips, washed with PBS, fixed with 4% paraformaldehyde for 10 min at 37 °C, and permeabilized with 1% Triton X-100 in PBS for 10 min. Between each steps, cells were rinsed three times with PBS. Cells were blocked with 1% bovine serum albumin (BSA) in PBS for 1 h at room temperature. For cellular localization of LC3 protein, cells were incubated with LC3A/B conjugated with Alexa Fluor 488 antibody diluted in blocking buffer overnight at 4 °C. Differentiated C2C12 cells were incubated with Texas Red X-Phalloidin (1:150, Invitrogen, Carlsbad, CA, USA) for 1 h. Cell nuclei were visualized with DAPI (NucBlue Fixed Cell ReadyProbes Reagent, Invitrogen).

Confocal images were acquired with Leica TCS SP8 confocal microscope (Leica, Wetzlar, Germany) and LAS X 3.5.5.19976 software (Leica, Wetzlar, Germany). Nonspecific binding of the secondary antibodies was checked in control experiments (not shown).

### 2.5. LysoTracker Deep Red Staining

Cells were grown on glass coverslips, washed with PBS, and stained with LysoTracker Deep Red (Thermo Fisher Scientific, Walthman, MA, USA) using a working concentration of 100 nM for 30 min and fixed in 4% paraformaldehyde for 10 min at 37 °C.

Confocal images were acquired with Leica TCS SP8 confocal microscope and LAS X 3.5.5.19976 software.

### 2.6. SDS-PAGE and Western Blotting

Cells were washed with PBS and lysed in lysis buffer (50 mM Tris, pH 8, 150 mM NaCl, 1% Triton X-100, 0.5% sodium deoxycholate, 0.1% SDS, 1 mM EDTA, 1 mM Na_3_VO_4_, 1 mM NaF, 1 mM PMSF, protease inhibitor cocktail) on ice and boiled with 5x SDS sample buffer (310 mM Tris-HCl, pH 6.8, 50% glycerol, 10% SDS, 100 mM DTT, 0.01% bromophenol blue) and 2-mercaptoethanol. Protein extracts were separated by SDS polyacrylamide gels and transferred onto nitrocellulose membranes. Membranes were blocked with 5% BSA in TBS_Tween_ for 1 h at room temperature and incubated with primary antibodies overnight at 4 °C. Membranes were probed with the respective peroxidase-conjugated secondary antibody. Signals were visualized by enhanced chemiluminescence reaction and captured by ChemiDoc Touch Imaging System (Bio-Rad, Hercules, CA, USA). Bands were quantified by densitometry using ImageJ software [33] and densitometry data were analyzed by statistical methods. Antibodies used in this study are shown in Table 2.

### 2.7. Total RNA Preparation, Reverse Transcription-coupled quantitative PCR (RT-qPCR)

Total RNA was prepared using TRIzol reagent (Invitrogen, Carlsbad, CA, USA) according to the manufacturer’s instructions. Then, 2 µg of total RNA was used for reverse transcription using a high-capacity cDNA reverse transcription kit (Applied Biosystem, Foster City, CA, USA). Diluted cDNA was used for qPCR reaction. Primers are listed in Table 3. Expression was normalized to the geometric mean of murine 34B4 and cyclophilin.

### 2.8. Electron Microscopy (EM)

Cell samples were processed for electron microscopic investigation similarly to [34]. Cells were fixed in 3% glutaraldehyde dissolved in 0.1 M cacodylate buffer (pH 7.4) containing 5% sucrose for 6 h at room temperature. After washing in 0.1 M cacodylate buffer (pH 7.4), cells were post-fixed in 1% osmium tetroxide dissolved in 0.1 M cacodylate buffer (pH 7.4) for 1 h. Then, cells were dehydrated with a graded ethanol series. Samples were embedded into DurcupanACM resin (Sigma-Aldrich). Ultrathin sections were cut with Leica Ultracut UCT Ultramicrotome, collected on Formvar-coated single-slot grids, and counterstained using uranyl acetate and Reynolds lead citrate. Sections were examined with a JEOL 1010 transmission electron microscope (JEOL Ltd., Akishima, Tokyo, Japan) and photographed with an Olympus Veleta CCD camera (Olympus, Sinjuku, Tokyo, Japan).

Morphometric assessment was accomplished using the ImageJ software. EM pictures of at least 45 different cells of each group were analyzed and cytosolic electron-dense particles were counted.

### 2.9. Statistical Analysis

LC3 or LysoTracker-positive vesicles were counted using ImageJ software. All numerical data are presented as the average ± SEM unless otherwise stated. For numerical values, significance between groups was analyzed by paired, two-tailed Student’s *t*-test. For multiple comparisons we used a one-way ANOVA test followed by Dunnett’s honestly significance (HSD) or Tukey’s post hoc test, as indicated in figure captions. The *n* number in the figure legends denotes the number of biological replicates.

## 3. Results

### 3.1. Silencing of PARP2 Induces Autophagy in C2C12 Cells

**Figure 1:**
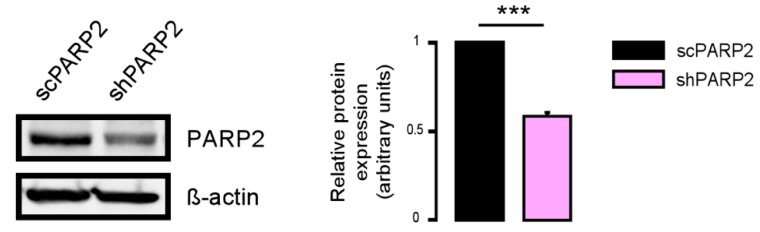
Validation of PARP2 silencing in stably-transfected C2C12 cells. PARP2 expression was assessed in scPARP2 and shPARP2 cells by Western blotting (*n* = 3). *** represents statistically significant differences between the scPARP2 and shPARP2 cells at *p* < 0.001.

**Figure 2:**
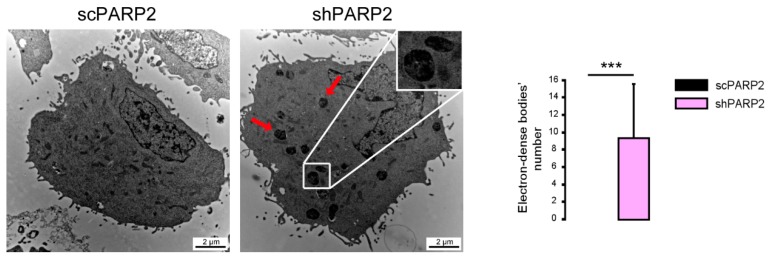
Cytosolic electron-dense particles appear in PARP2-silenced cells. scPARP2 and shPARP2 C2C12 cells were analyzed by electron microscopy (*n* = 1, counted cells: 50/50). Red arrows and the insert picture show the cytosolic electron-dense particles in shPARP2 cells, which were absent in scPARP2 cells. Cytosolic electron-dense particles were counted in cells and data was plotted. *** represents statistically significant differences between the scPARP2 and shPARP2 cells at *p* < 0.001. Average ± SD is plotted. As cytosolic electron-dense bodies were absent in the scPARP2 cells, the value for the chart is 0 with no standard deviation.

As the model system, we chose C2C12 cells in which PARP2 was silenced (shPARP2) and their isogenic control line (scPARP) was transfected with control (non-specific) shRNA sequence [31,32] (Figure 1). These cells were subjected to electron microscopy analysis. We were surprised to find cytosolic electron-dense particles exclusively in the shPARP2 C2C12 cells (Figure 2) that looked like late-stage autophagic vesicles (that is, autophagosomes that underwent fusion with late endosomes or lysosomes, with cytoplasmic cargo still recognizable in their lumen).

**Figure 3:**
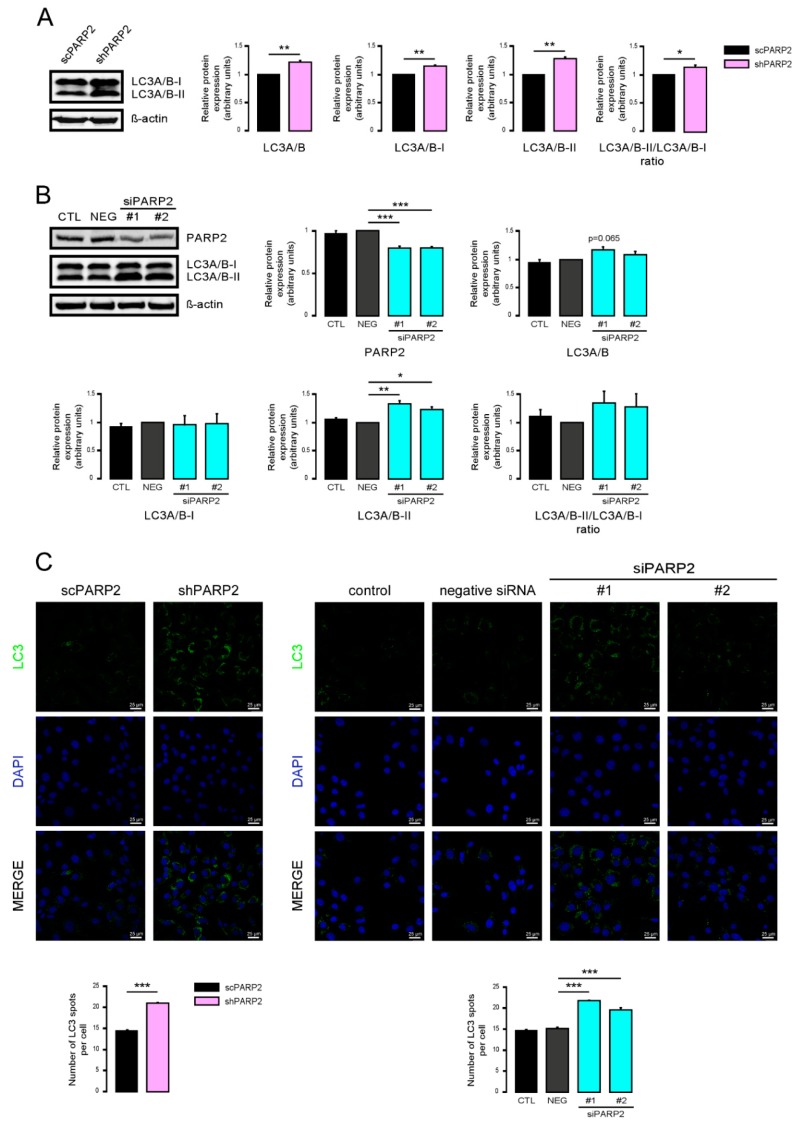
Silencing of PAPR2 increases the level of LC3. (**A**) In scPARP2 and shPARP2 C2C12 cells, LC3 expression was analyzed by Western blotting (*n* = 3). (**B**) PARP2 was transiently silenced in C2C12 cells using two different siRNAs (*n* = 3). Cells were transfected with siRNAs for 48 h, then PARP2 and LC3 levels were determined by Western blotting. (**C**) LC3+DAPI immunofluorescence was performed in scPARP2 and shPARP2 C2C12 and in C2C12 cells where PARP2 was transiently silenced (*n* = 3). Alexa Fluor 488-linked LC3 specific antibody was used and the nuclei were visualized using DAPI and vesicles were counted. Representative images are presented in the figure. *, **, and *** represent statistically significant differences between the indicated groups at *p* < 0.05, *p* < 0.01 and, *p* < 0.001, respectively. For the determination, ANOVA test was used followed by Dunnett’s post hoc test. NEG–Negative control, where cells were transfected with non-specific control siRNA.

**Figure 4:**
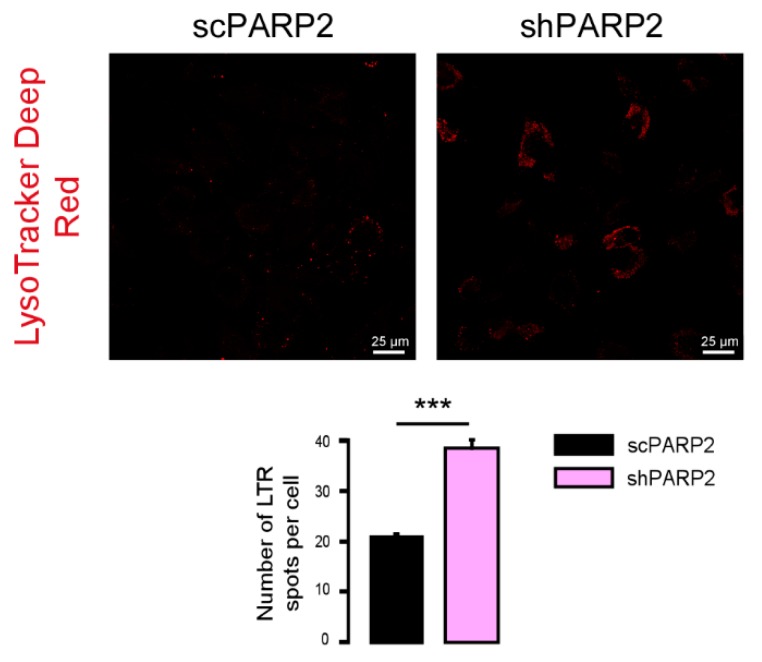
Silencing of PARP2 increases the number of acidic lysosomes. scPARP2 and shPARP2 C2C12 cells were stained with LysoTracker Deep Red (*n* = 3, counted cells: 100/100). LysoTracker Deep Red was assessed in confocal microscopy experiments and vesicles were counted. Representative images are presented in the figure. *** represent statistically significant differences between the indicated groups at *p* < 0.001.

To provide evidence that these vesicles were indeed of autophagic nature, we determined LC3 levels in scPARP2 and shPARP2 cells. LC3 levels were induced in the shPARP2 cells compared to the scPARP2 controls (Figure 3A), with a striking increase in the level of lipidated, autophagic membrane-associated LC3-II. Since the scPARP/shPARP2 C2C12 cell line pair was established years earlier, we performed transient silencing with siRNA molecules. Both PARP2-specific siRNA molecules efficiently reduced the expression of PARP2 and increased the level of lipidated LC3-II (Figure 3B). Finally, we assessed LC3 expression and distribution in immunofluorescence (IF) experiments that showed similar results to Western blotting: a striking increase in the number of strongly LC3-positive vesicles were found in PARP2-silenced cells compared to the respective controls (Figure 3C). As an alternative to LC3 staining, we charged scPARP2 and shPARP2 cells with LysoTracker that stains acidic vesicles, i.e., autolysosomes. Using LysoTracker we also observed a marked induction of punctate staining in the shPARP2 cell population (Figure 4).

**Figure 5:**
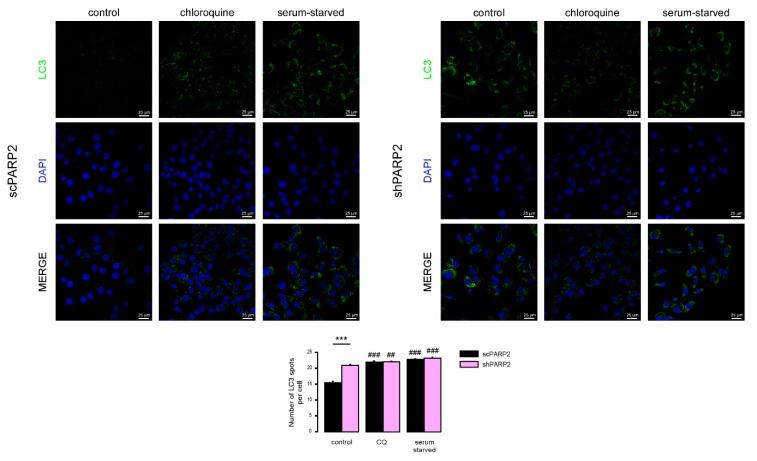
Chloroquine and serum starvation induce LC3 expression in scPARP2 and shPARP2 cells to the same extent. scPARP2 and shPARP2 C2C12 cells were treated with 25 µM chloroquine for 2 h or serum-starved for 12 h. LC3 expression was assessed in confocal microscopy experiments. Alexa Fluor 488-linked LC3 specific antibody was used and the nuclei were visualized using DAPI, vesicles were counted. Representative images are presented on the figure. ## and ### represent statistically significant differences between the control and chloroquine-treated or control and serum-starved cells at *p* < 0.01 or *p* < 0.001, respectively. *** represent statistically significant differences between the scPARP2 and shPARP2 cells at *p* < 0.001. For the determination of statistical significance ANOVA test was used followed by Tukey’s post hoc test.

We next assessed how the two cell lines respond to known modulators of autophagy, chloroquine (blocks the clearance of autophagic vesicles by neutralizing lysosomes) and serum starvation (increases the formation of autophagic vesicles). Both interventions led to an increased punctate LC3 signal, which was comparable in both cell lines (Figure 5). These data suggest that, while both cell lines respond to starvation by increased formation of autophagic vesicles, basal autophagic flux is partially impaired in PARP2 loss-of-function cells based on similar LC3 vesicle numbers seen in the two cell lines upon treatment with the autophagic degradation inhibitor chloroquine.

### 3.2. Induction of Autophagy Depends on the Induction of SIRT1 and the Inhibition of AMPK

**Figure 6:**
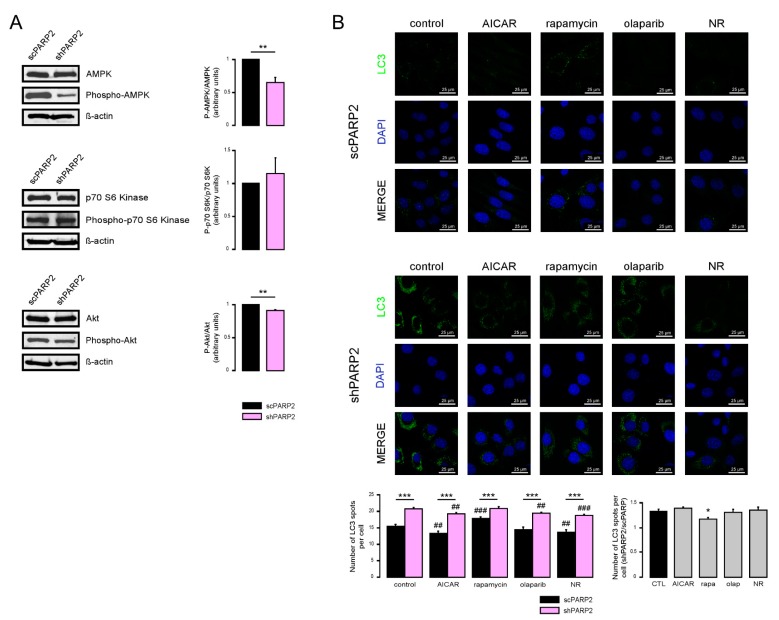
Inhibition of AMPK and cellular NAD^+^ level modulate autophagy in a PARP2-dependent fashion. (**A**) In scPARP2 and shPARP2 C2C12 cells, the change of AMPK, phospho-AMPK, p70 S6 kinase, phospho-p70 S6 kinase, Akt, and phospho-Akt expression were analyzed by Western blotting (*n* = 3). (**B**) scPARP2 and shPARP2 C2C12 cells were treated with 1 mM AICAR, 20 nM rapamycin (rapa), 1 µM olaparib (olap), or 500 µM nicotinamide-riboside (NR) for 24 h. LC3 expression was assessed in confocal microscopy experiments. Alexa Fluor 488-linked LC3 specific antibody was used and the nuclei were visualized using DAPI and vesicles were counted. Representative images are presented in the figure. ## and ### represent statistically significant differences between the control and treated cells at *p* < 0.01 and *p* < 0.001, respectively. *, ** and *** represent statistically significant differences between the scPARP2 and shPARP2 cells at *p* < 0.05, *p* < 0.01 and *p* < 0.001. For the determination of statistical significance, ANOVA test was used followed by Tukey’s post hoc test.

We assessed the function of a set of energy sensors in scPARP2 and shPARP2 cells and we found a deregulation of cellular energy sensors. mTORC1 activity, measured through assessing the phosphorylation of the p70 S6 kinase (Phospho-p70 S6 Kinase (Thr389)), did not change in the shPARP2 cells (Figure 6A). In contrast, AMPK activity, marked by the auto-phosphorylation of AMPK (Phospho-AMPKα (Thr172)), was profoundly reduced (Figure 6A). Finally, mTORC2 activity, assessed through the phosphorylation of Akt kinase (Phospho-Akt (Ser473)), showed a mild reduction (Figure 6A). Apparently, the silencing of PARP2 led to profound changes in cellular energy sensing.

Next, we assessed whether these changes are functional by using pharmacological modulators of the above energy sensors. The use of AICAR, an activator of AMPK, reduced the proportions of the high LC3 expression cells compared to the shPARP2 control cells (Figure 6B), suggesting that the suppression of AMPK activity has a causative role in the induction of autophagy. Rapamycin, an inhibitor of mTORC1, did not change the proportions of LC3 positivity (Figure 6B). Olaparib, a PARP inhibitor, in line with the previous literature [17,18,19], decreased baseline LC3 expression, but was unable to prevent increases in LC3 expression in the shPARP2 cells (Figure 6B). Nicotinamide riboside (NR) decreased LC3 expression in scPARP2 cells and prevented increases in LC3 expression in shPARP2 cells (Figure 6B).

**Figure 7:**
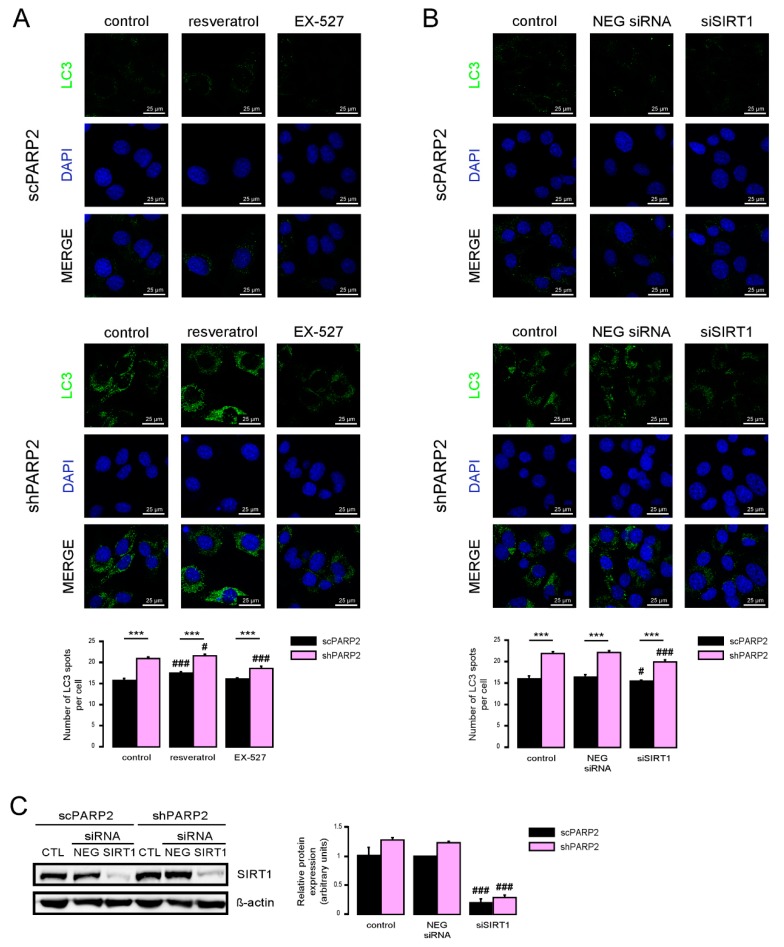
SIRT1 activation is a key determinant of the PARP2-mediated inhibition of autophagy. (**A**) scPARP2 and shPARP2 C2C12 cells were treated with 50 µM resveratrol or 25 µM EX-527 for 24 h (*n* = 3). LC3 expression was assessed by confocal microscopy. Alexa Fluor 488-linked LC3 specific antibody was used and the nuclei were visualized using DAPI and vesicles were counted. Representative images are presented on the figure. (**B**,**C**) SIRT1 was transiently silenced in scPARP2 and shPARP2 C2C12 cells using siRNA targeting SIRT1 (*n* = 3). Cells were transfected with siRNA for 48 h, then LC3 expression was assessed in confocal microscopy experiments and the protein level of SIRT1 was detected by Western blotting. Alexa Fluor 488-inked LC3 specific antibody was used and the nuclei were visualized using DAPI. Representative images are presented in the figure. Cells were scored as described in Materials and Methods. # and ### represent statistically significant differences between the control and treated cells at *p* < 0.05 and *p* < 0.001, respectively. *** represent statistically significant differences between the scPARP2 and shPARP2 cells at *p* < 0.001. For the determination of statistical significance, ANOVA test was used followed by Tukey’s post hoc test.

SIRT1 activation can induce autophagy [35,36] and silencing of PARP2 was shown to induce SIRT1 expression [5,31,37]. Therefore, we assessed whether SIRT1 could be implicated in the upregulation of autophagy. We activated SIRT1 using resveratrol [38] and inhibited its enzymatic activity using EX-527 [39]. Resveratrol induced LC3 expression both in the scPARP2 and shPARP2 cells (Figure 7A). EX-527 treatment did not alter LC3 expression in scPARP2 C2C12 cells, while it reduced LC3 expression in shPARP2 C2C12 cells (Figure 7A).

We also assessed if silencing of SIRT1 can phenocopy the effects of EX-527. We silenced SIRT1 transiently in C2C12 cells using siRNA (Figure 7B). siRNA silencing of SIRT1 decreased LC3 expression in shPARP2 C2C12 cells (Figure 7C).

**Figure 8:**
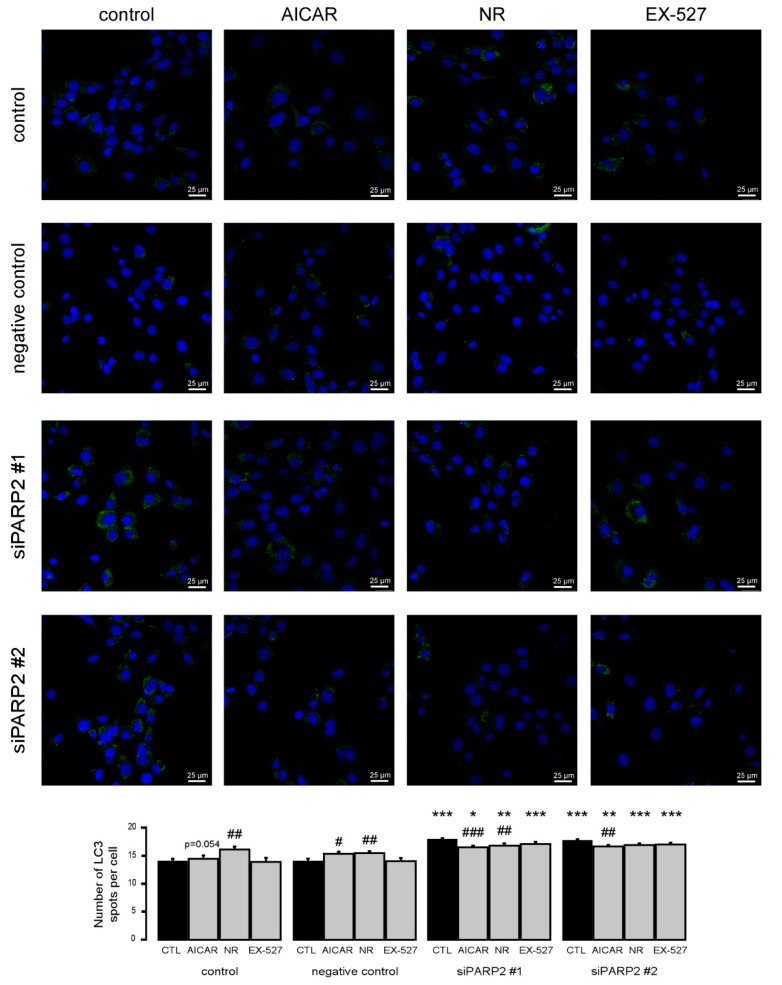
Increased autophagy upon acute PARP2 silencing. PARP2 was transiently silenced in C2C12 cells using siRNA targeting PARP2 (*n* = 3). Cells were transfected with siRNA for 48 h, treated with 1 mM AICAR, 500 µM nicotinamide-riboside (NR), and 25 µM EX-527 for 24 h, then LC3 expression was assessed by confocal microscopy. Alexa Fluor 488-linked LC3 specific antibody was used and the nuclei were visualized using DAPI and vesicles were counted. #, ## and ### represent statistically significant differences between the control and treated cells at *p* < 0.05, *p* < 0.01 or *p* < 0.001, respectively. *, **, and *** represent statistically significant differences between the scPARP2 and shPARP2 cells at *p* < 0.05, *p* < 0.01, and *p* < 0.001, respectively. For the determination of statistical significance, ANOVA test was used followed by Tukey’s post hoc test.

To complement the pharmacological approach, we treated C2C12 cells that were transiently transfected with PARP2 siRNA with the previously identified inhibitors of PARP2-induced autophagy (AICAR, nicotinamide-riboside, and EX-527). All agents were able to inhibit increases in LC3 expression similar to previous observations (Figure 8).

### 3.3. The Number of LC3-Positive Vesicles Increase in Primary Murine Embryonic Fibroblasts Upon the Genetic Deletion of PARP2

**Figure 9:**
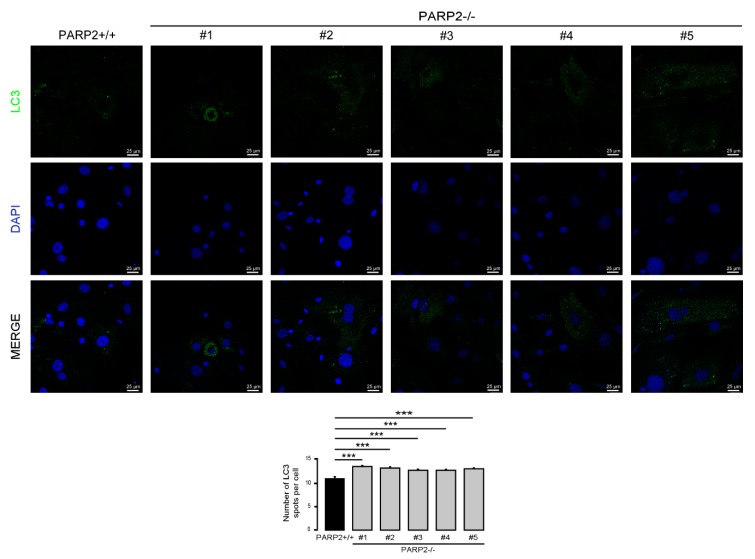
The number of LC3-positive vesicles in primary PARP2 knockout MEF cells. A total of 15,000 cells from the indicated primary MEF cells were seeded and were stained for Alexa Fluor 488-linked LC3 specific antibody and the nuclei with DAPI. Vesicles were counted. Representative images are presented in the figure. *** represents statistically significant differences between the PARP2^+/+^ and PARP2^−^^/−^ MEF cells at *p* < 0.001. For the determination of statistical significance, ANOVA test was used followed by Dunnett’s post hoc test. The different numbers represent different MEF clones.

In C2C12 cells, the efficiency of the silencing of PARP2 was around 50%. Therefore, we assessed primary murine embryonic fibroblasts (MEFs) derived from PARP2 knockout mice [32,40], where PARP2 protein was completely absent. The MEFs used in the current study were from het-to-het breeding-derived male embryos; MEFs were generated for a previous study [32]. In these cells we assessed the number of LC3-positive vesicles. In good agreement with previous observations, the number of LC3-positive vesicles increased in the PARP2^−/−^ MEF cells compared to their PARP2^+/+^ counterparts (Figure 9).

### 3.4. The Activity of PARP2 Plays Role in Mediating Autophagy

**Figure 10:**
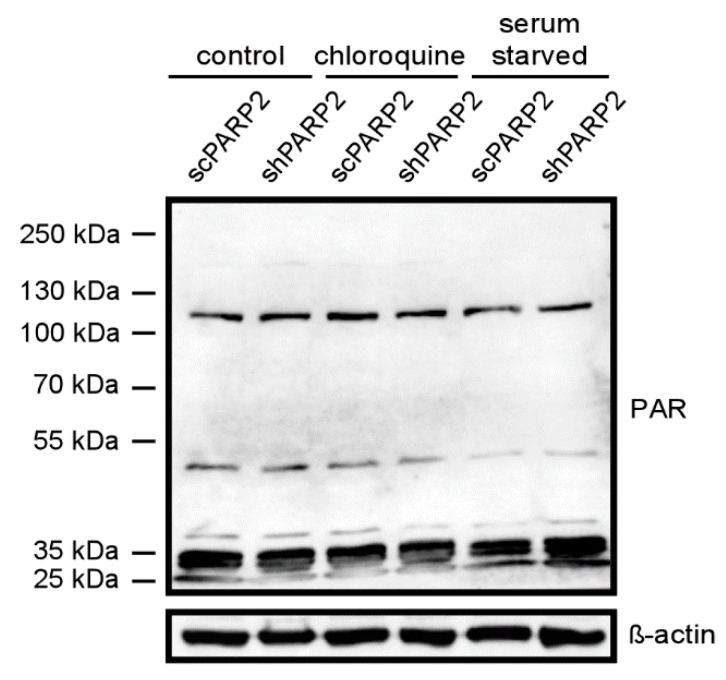
The silencing of PARP2 modulates cellular PARylation under chloroquine treatment and fasting. scPARP2 and shPARP2 cells were treated with chloroquine (25 µM 2 h) or were fasted for 12 h. Then cells were lysed and lysates were separated by PAGE and were subjected to Western blotting using anti-PAR antibody. Experiments were repeated three times; a representative blot is shown.

Subsequently, we assessed whether the activity of PARP2 could play a role in the regulation of autophagy. PARP2 is responsible for ~10–15% of total cellular PARP activity [5,6]. We assessed total PARP activity in cells by assessing PARylation pattern in cellular lysates (Figure 10). Chloroquine treatment induced PARylation (similarly as in [22]) and fasting reduced PARylation [41]. Silencing of PARP2 did not change the PARylation pattern in non-treated cells. The silencing of PARP2 reduced chloroquine-induced PARylation and antagonized fasting-induced suppression of PARylation, which altogether suggests a role for the enzymatic activity of PARP2 in the regulation of autophagy.

**Figure 11:**
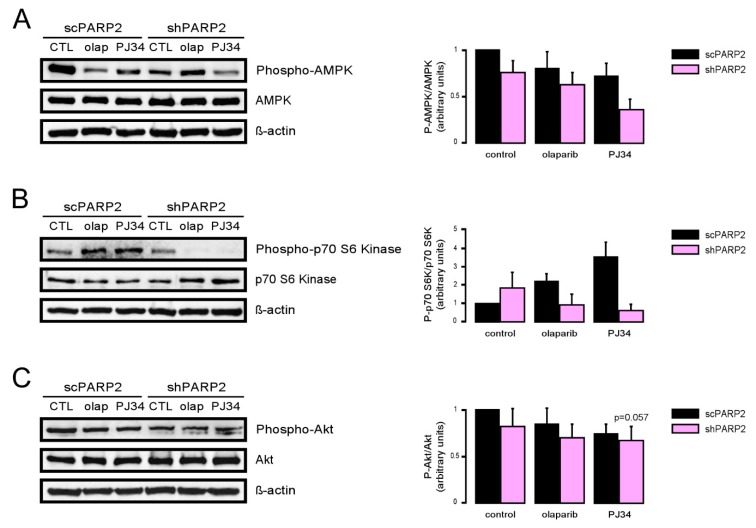
PARP2 enzymatic activity modulates the energy sensors involved in the regulation of autophagy. A total of 200,000 scPARP2 and shPARP2 C2C12 cells were seeded in 6-well plates that were treated with olaparib (olap, 1 µM) and PJ34 (3 µM) for 24 h. Cellular proteins were separated by SDS-PAGE and were blotted and probed with the antibodies indicated on panels (**A**–**C**). Bands were subjected to densitometry. Experiments were repeated three times; a representative blot is shown.

As PARP activity changed as a function of the reduction of PARP2 protein content, we assessed whether pharmacological PARP inhibition may impact the changes to cellular energy sensors. As the currently used PARP inhibitors cannot discriminate between PARP1 and PARP2 [42,43], we tried to assess whether and how PARP2 activity contributes to the regulation of autophagy by applying PARP inhibitors (olaparib and PJ34) onto scPARP2 and shPARP2 cells (similar setup as in [44]). The silencing of PARP2 decreased AMPK activity, as marked by the lower phosphorylation level of AMPK. In scPARP2 C2C12 cells, AMPK activity was suppressed by PARP inhibition, while in shPARP2 cells PARP inhibition did not drastically further decrease AMPK activity (Figure 11A). The silencing of PARP2 did not alter mTORC1 activity, marked by the phosphorylation of p70 S6 kinase. However, when PARP inhibitors were applied, p70 S6 kinase phosphorylation levels were induced in the scPARP2 cells. In stark contrast to that, PARP inhibition in the shPARP2 cells led to the dephosphorylation of p70 S6 kinase (Figure 11B). Finally, we assessed the activity of mTORC2 through measuring Akt phosphorylation. The silencing of PARP2 decreased Akt phosphorylation, indicative of a decrease in mTORC2 activity. Inhibition of PARP activity decreased Akt phosphorylation in the control and scPARP2 cells, while PARP inhibition did not further decrease Akt phosphorylation (Figure 11C). Taken together, PARP2 activity is involved in the regulation of the cellular energy sensor system and, in particular, the systems regulating autophagy.

### 3.5. Silencing of PARP2 Affects the Differentiation of C2C12 Myoblasts

**Figure 12:**
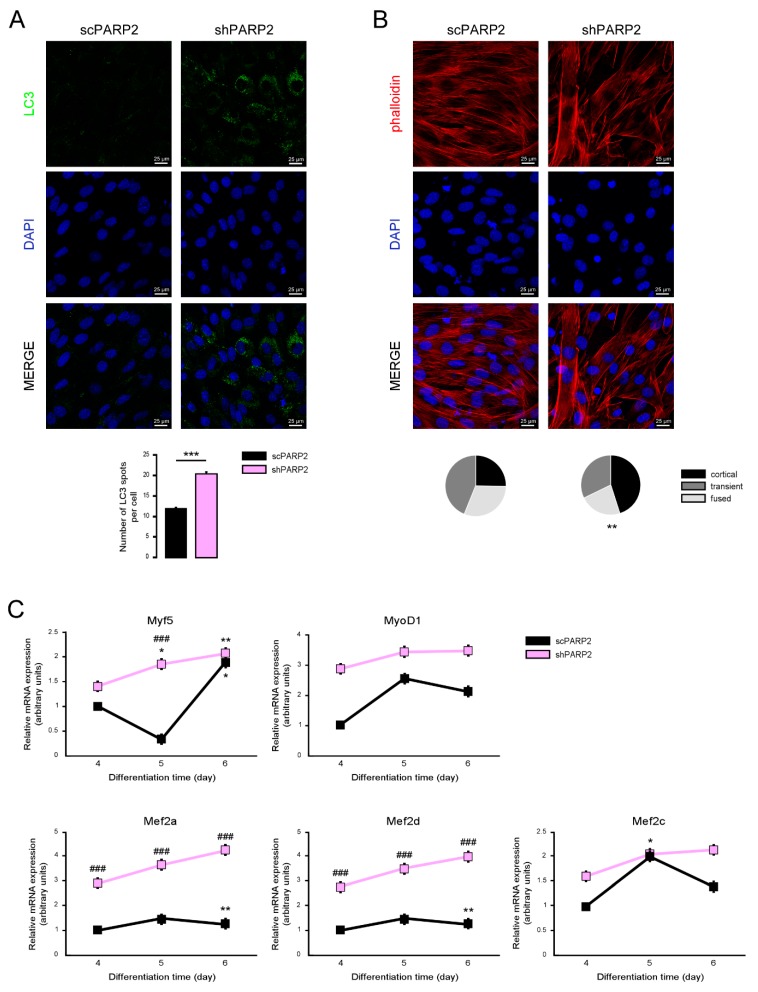
Silencing of PARP2 supports myogenic differentiation. A total of 20,000 scPARP2 and shPARP2 C2C12 cells were seeded in 24-well plates that were differentiated for 4 days then (**A**) LC3-positive vesicles were assessed by immunofluorescence, (**B**) actin cytoskeleton was stained by Texas Red-X Phalloidin+DAPI, and (**C**) the expression of a set of myogenic genes were assessed by RT-qPCR. On panel B 100/100 cells were scored for the structure of actin cytoskeleton as diffuse, transient, and strong cortical staining. *** represent statistical difference between scPARP2 and shPARP2 cells at *p* < 0.001 on panel A. ** represent statistical difference between scPARP2 and shPARP2 cells in terms of actin morphology at *p* < 0.01 on panel B. In panel C, ### represents statistically significant differences between the scPARP2 and shPARP2 cells at *p* < 0.001. * and ** represent statistically significant differences between the control and differentiated cells at *p* < 0.05 and *p* < 0.01, respectively. For the determination of statistical significance, ANOVA test was used followed by Tukey’s post hoc test on panels A and C, while on panel B chi square test was applied.

The importance of PARPs were demonstrated in decision making towards differentiation [9,45,46,47,48,49,50,51,52], including skeletal muscle differentiation [53,54,55,56]. The knockout of PARP2 induced a conversion to more type I and oxidative type II fibers [31]. Furthermore, autophagy was implicated in muscular differentiation and sarcopenia [57,58], making it likely that the silencing of PARP2 may affect the differentiation of C2C12 cells. The dramatic increase in LC3-positive vesicles was maintained in differentiated shPARP2 C2C12 cells (Figure 12A). We assessed the morphology of the differentiated C2C12 cells and found that the cortical actin staining was more pronounced in shPARP2 cells compared to scPARP2 cells (Figure 12B). Finally, we assessed the expression of different myogenic differentiation markers (Myf5, MyoD1, Mef2a, Mef2d and Mef2c) on day 4, day 5, and day 6 of differentiation. All markers showed higher expression in the shPARP2 cells compared to scPARP2 cells (Figure 12C).

## 4. Discussion

Herein, we showed that the genetic or pharmacological silencing of PARP2 induces the number of autophagic vesicles in cellular models through inhibiting AMPK and inducing SIRT1 activity. Two known treatments that increase autophagic vesicle numbers, chloroquine and serum starvation, increased punctate LC3 signal to levels that are comparable in both cell lines. This suggests that both cell lines respond to starvation by increased formation of autophagic vesicles and that basal autophagic flux is partially impaired in PARP2 loss-of-function cells. It is of note that in the C2C12 cells the reduction of PARP2 expression was around 50%, which limits the applicability of our findings. Nevertheless, we showed that the number of LC3-positive increases in PARP2 knockout MEF cells too, validating our findings in C2C12 cells.

PARP2 was originally described as a DNA repair protein. However, recent investigations have shed light on a strong connection between PARP2 and metabolism [4,59]. PARP2 can be activated by irregular DNA forms [60,61] and by binding to RNA [3]. Besides these, its activity can be modulated by posttranslational modifications, such as acetylation, PARylation [62], or by controlling its expression by lipid biomolecules [63,64] or flavonoids [65], and through mediating its subcellular localization by serum starvation [66]. PARP2 was linked to DNA repair [6,40], thymo- and hematopoiesis [46,47,48], as well as, metabolism [67,68]. We extended the list of these functions by adding its effects on autophagy.

PARP1 and PARP10 was already implicated in the regulation of autophagy. PARP10 was shown to interact with ubiquitin receptor p62 [16], suggesting a role for PARP10 in selective autophagy. PARP1 activation is needed for the appropriate initiation of fasting-induced autophagy [18]. Starvation induces DNA damage [18] and, hence, PARP1 activity [18,41], which is an initiating step for autophagy. PARP1-mediated induction of autophagy requires the coordinated activation of AMPK [18,69,70,71,72]. In fact, PARylation and nuclear export of AMPK are necessary for the initiation of autophagy [19]. There is also contradicting evidence, however, where PARP inhibition induced autophagy or mitophagy [73,74]. SIRT1 activation was also shown to influence autophagy [13,14,15]. However, the involvement of the PARP1-SIRT1 axis [31,41,75] is also controversial [76,77]. These controversies in the role of PARP1 suggest the involvement of PARP2 in these model systems.

We showed that PARP2 controls autophagy through rearranging the cellular energy sensor system. The genetic inhibition of PARP2 blocked AMPK activity, while pharmacological activation of AMPK partially blocked the PARP2-dependent induction of autophagy. These findings suggest a causative role of AMPK inhibition in the induction of autophagy. This finding is intriguing as previous literature described AMPK as an inductor of autophagy [18,69,70,71,72,78]. Somewhat surprisingly, we did not observe changes in the activity of mTORC1 upon PARP2 silencing. Nevertheless, the activity of the mTORC2 complex was downregulated. mTORC1-independent and mTORC2-dependent forms of autophagy were already reported [79,80,81,82]. The actual molecular link between PARP2 and energy sensors is yet unknown.

A potent NAD^+^ precursor molecule, nicotinamide riboside [83] was able to revert PARP-2-induced autophagic vesicle accumulation. NAD^+^ availability or NR supplementation promotes autophagy in various models [84,85]. In fact, NAD^+^ availability improves the elimination of damaged proteins [85]. PARP2 is an NAD^+^-dependent enzyme [2,5,6] and, therefore, it is likely that NR supports the activity of PARP2 through enhancing cellular NAD^+^ levels. This scenario is likely as the enzymatic activity of PARP2 is required for its DNA-repair and non-repair functions [2,44,86]. Nevertheless, it should be stated that SIRT1 independent pathways also exist upon the ablation of PARP2 that modulate autophagy, pathways that probably involve the activation of the above-discussed energy sensor pathways.

In line with the above, SIRT1 inhibition was the most dominant in reducing the accumulation of autophagic vesicles in shPARP2 cells. Previous studies [5,31,37,56,64] established the molecular link between SIRT1 and PARP2. PARP2 is a repressor of the promoter of SIRT1 and, hence, the ablation of PARP2 induces SIRT1 expression and SIRT1 activity [31]. Furthermore, as SIRT1 and PARP2 are both NAD^+^-dependent enzymes that compete for the same NAD+ pool [2,5,6,37,75,87], the genetic inactivation of PARP2 can induce NAD^+^ levels and contribute to the induction of SIRT1 [37]. These data link autophagy to cellular NAD^+^ homeostasis.

What physiological or pathological changes can influence PARP2 expression when the regulatory effects of PARP2 may impact on autophagy? To date, the effects modulating PARP2 expression had not been assessed in detail. Nevertheless, there are studies in the literature where the pathology discussed is related to autophagy induction. Sun and colleagues [66] showed that serum deprivation, a known inducer of autophagy, can decrease PARP2 expression. Furthermore, there are lipid species and drugs that can modulate PARP2 expression [63]. Finally, PARP2 depletion is partially protective in neurodegenerative diseases that are related to the deregulation of autophagy [88,89]. As a logical continuation to this list, the silencing of PARP2 supported the differentiation of myoblasts to myofibers, at least in part, through the modulation of autophagy and, in line with that, the depletion of PARP2 was protective against cancer cachexia and muscle wasting [56].

PARP2 represents a novel link between DNA repair and autophagy machinery [90,91]. Silencing of PARP2 blocks the processing of the autophagic vesicles, suggesting its involvement in the progression of autophagy. However, the exact molecular role of PARP2 remains to be elucidated.

## Figures and Tables

**Table 1:** The source of key chemicals used in the study.

Chemical	Company	Catalog Number	Concentration	Length of Treatment
chloroquine	Sigma-Aldrich	C6628	25 µM	2 h
AICAR	Santa Cruz BT	sc-200659A	1 mM	24 h
rapamycin	Cayman Chemical	13346	20 nM	24 h
olaparib	Selleckchem	S1060	1 µM	24 h
NR	ChromaDex	-	500 µM	24 h
resveratrol	Sigma-Aldrich	R5010	50 µM	24 h
EX-527	Selleckchem	S1541	25 µM	24 h
PJ34	Sigma-Aldrich	P4365	3 µM	24 h

AICAR—5-Aminoimidazole-4-carboxamide ribonucleotide, NR—nicotinamide-riboside.

**Table 2:** Antibodies used in the study.

Antibody	Company	Dilution
LC3A/B Alexa Fluor 488 Conjugate	Cell Signaling Technology, 13082	1:50
LC3A/B	Cell Signaling Technology, 12741	1:1000
PARP2	Enzo Life Sciences, ALX-210-899-R100	1:2000
SIRT1	EMD Millipore, 07-131	1:1000
AMPKα	Cell Signaling Technology, 5832	1:1000
Phospho-AMPKα (Thr172)	Cell Signaling Technology, 2535	1:1000
p70 S6 Kinase	Sigma-Aldrich, SAB4502691	1:1000
Phospho-p70 S6 Kinase (Thr389)	Cell Signaling Technology, 9205	1:1000
Akt	Cell Signaling Technology, 9272	1:1000
Phospho-Akt (Ser473)	Cell Signaling Technology, 4060	1:1000
Poly(ADP-ribose)	Enzo Life Sciences, BML-SA216-0100	1:1000
Anti-mouse IgG, HRP-linked	Sigma-Aldrich, A9044	1:2000
Anti-rabbit IgG, HRP-linked	Cell Signaling Technology, 7074	1:2000
Anti-β-actin-Peroxidase	Sigma-Aldrich, A3854	1:20,000

**Table 3:** Primers used in the study.

Gene Name	Forward Primer	Reverse Primer
Myf5	GACACAGCTTCCCTCTCTCCAG	ACGTATTCTGCCCAGCTTGTCT
MyoD1	GCTTTGAGAGATCGACTGCAGC	TGTCCTTTCTTTGGGGCTGGAT
Mef2a	CTCCCCGTGATAGAATGACCCC	GGTCACTGCCATCATAGGAGCT
Mef2d	GTGTTTACAAGGGATCAGCGCC	AGAGCTCCAAATGTGAAGCCCT
Mef2c	ACGATTCAGTAGGTCACAGCCC	CTGTTATGGCTGGACACTGGGA
cyclophilin	TGGAGAGCACCAAGACAGACA	TGCCGGAGTCGACAATGAT
36B4	AGATTCGGGATATGCTGTTGG	AAAGCCTGGAAGAAGGAGGTC

## Data Availability

All primary data is uploaded to https://figshare.com/s/6dde52090d39ea56eddb (DOI: 10.6084/m9.figshare.8832491).

## References

[1] Bai P. (2015). Biology of Poly(ADP-Ribose) Polymerases: The Factotums of Cell Maintenance. Mol. Cell.

[2] Ame J.C., Rolli V., Schreiber V., Niedergang C., Apiou F., Decker P., Muller S., Hoger T., Menissier-de Murcia J., de Murcia G. (1999). PARP-2, A novel mammalian DNA damage-dependent Poly(ADP-Ribose) polymerase. J. Biol. Chem..

[3] Leger K., Bar D., Savic N., Santoro R., Hottiger M.O. (2014). ARTD2 activity is stimulated by RNA. Nucleic Acids Res..

[4] Szanto M., Brunyánszki A., Kiss B., Nagy L., Gergely P., Virag L., Bai P. (2012). Poly(ADP-Ribose) polymerase-2: Emerging transcriptional roles of a DNA repair protein. Cell Mol. Life Sci..

[5] Szanto M., Rutkai I., Hegedus C., Czikora A., Rozsahegyi M., Kiss B., Virag L., Gergely P., Toth A., Bai P. (2011). Poly(ADP-Ribose) polymerase-2 depletion reduces doxorubicin-induced damage through SIRT1 induction. Cardiovasc. Res..

[6] Schreiber V., Ame J.C., Dolle P., Schultz I., Rinaldi B., Fraulob V., Menissier-de Murcia J., de Murcia G. (2002). Poly(ADP-Ribose) polymerase-2 (PARP-2) is required for efficient base excision DNA repair in association with PARP-1 and XRCC1. J. Biol. Chem..

[7] Bai P., Nagy L., Fodor T., Liaudet L., Pacher P. (2015). Poly(ADP-Ribose) polymerases as modulators of mitochondrial activity. Trends Endocrinol. Metab..

[8] Gupte R., Liu Z., Kraus W.L. (2017). PARPs and ADP-ribosylation: Recent advances linking molecular functions to biological outcomes. Genes Dev..

[9] Ryu K.W., Nandu T., Kim J., Challa S., DeBerardinis R.J., Kraus W.L. (2018). Metabolic regulation of transcription through compartmentalized NAD(+) biosynthesis. Science.

[10] Levine B., Kroemer G. (2019). Biological Functions of Autophagy Genes: A Disease Perspective. Cell.

[11] Bhattacharjee A., Szabo A., Csizmadia T., Laczko-Dobos H., Juhasz G. (2019). Understanding the importance of autophagy in human diseases using Drosophila. J. Genet. Genom..

[12] Klionsky D.J., Abdelmohsen K., Abe A., Abedin M.J., Abeliovich H., Acevedo Arozena A., Adachi H., Adams C.M., Adams P.D., Adeli K. (2016). Guidelines for the use and interpretation of assays for monitoring autophagy (3rd ed.). Autophagy.

[13] Martinez-Outschoorn U.E., Peiris-Pages M., Pestell R.G., Sotgia F., Lisanti M.P. (2017). Cancer metabolism: A therapeutic perspective. Nat. Rev. Clin. Oncol..

[14] Quan W., Lee M.S. (2013). Role of autophagy in the control of body metabolism. Endocrinol. Metab. (Seoul).

[15] Singh R., Cuervo A.M. (2011). Autophagy in the cellular energetic balance. Cell Metab..

[16] Kleine H., Herrmann A., Lamark T., Forst A.H., Verheugd P., Luscher-Firzlaff J., Lippok B., Feijs K.L., Herzog N., Kremmer E. (2012). Dynamic subcellular localization of the mono-ADP-ribosyltransferase ARTD10 and interaction with the ubiquitin receptor p62. Cell Commun. Signal.

[17] Munoz-Gamez J.A., Rodriguez-Vargas J.M., Quiles-Perez R., Aguilar-Quesada R., Martin-Oliva D., de Murcia G., Menissier de Murcia J., Almendros A., Ruiz de Almodovar M., Oliver F.J. (2009). PARP-1 is involved in autophagy induced by DNA damage. Autophagy.

[18] Rodriguez-Vargas J.M., Ruiz-Magana M.J., Ruiz-Ruiz C., Majuelos-Melguizo J., Peralta-Leal A., Rodriguez M.I., Munoz-Gamez J.A., de Almodovar M.R., Siles E., Rivas A.L. (2012). ROS-induced DNA damage and PARP-1 are required for optimal induction of starvation-induced autophagy. Cell Res..

[19] Rodriguez-Vargas J.M., Rodriguez M.I., Majuelos-Melguizo J., Garcia-Diaz A., Gonzalez-Flores A., Lopez-Rivas A., Virag L., Illuzzi G., Schreiber V., Dantzer F. (2016). Autophagy requires poly(adp-ribosyl)ation-dependent AMPK nuclear export. Cell Death Differ..

[20] Santiago-O’Farrill J.M., Weroha S.J., Hou X., Oberg A.L., Heinzen E.P., Maurer M.J., Pang L., Rask P., Amaravadi R.K., Becker S.E. (2019). Poly(adenosine diphosphate ribose) polymerase inhibitors induce autophagy-mediated drug resistance in ovarian cancer cells, xenografts, and patient-derived xenograft models. Cancer.

[21] Liu Y., Song H., Song H., Feng X., Zhou C., Huo Z. (2019). Targeting autophagy potentiates the anti-tumor effect of PARP inhibitor in pediatric chronic myeloid leukemia. AMB Express.

[22] Zai W., Chen W., Han Y., Wu Z., Fan J., Zhang X., Luan J., Tang S., Jin X., Fu X. (2019). Targeting PARP and autophagy evoked synergistic lethality in hepatocellular carcinoma. Carcinogenesis.

[23] Xu L., Liu J., Chen Y., Yun L., Chen S., Zhou K., Lai B., Song L., Yang H., Liang H. (2017). Inhibition of autophagy enhances Hydroquinone-induced TK6 cell death. Toxicol. In Vitro.

[24] Liu W., Dai N., Wang Y., Xu C., Zhao H., Xia P., Gu J., Liu X., Bian J., Yuan Y. (2016). Role of autophagy in cadmium-induced apoptosis of primary rat osteoblasts. Sci. Rep..

[25] Zhou Z.R., Zhu X.D., Zhao W., Qu S., Su F., Huang S.T., Ma J.L., Li X.Y. (2013). Poly(ADP-Ribose) polymerase-1 regulates the mechanism of irradiation-induced CNE-2 human nasopharyngeal carcinoma cell autophagy and inhibition of autophagy contributes to the radiation sensitization of CNE-2 cells. Oncol. Rep..

[26] Kim J., Lim W., Kim S., Jeon S., Hui Z., Ni K., Kim C., Im Y., Choi H., Kim O. (2014). Photodynamic therapy (PDT) resistance by PARP1 regulation on PDT-induced apoptosis with autophagy in head and neck cancer cells. J. Oral Pathol. Med..

[27] Wang X., Tu W., Chen D., Fu J., Wang J., Shao C., Zhang J. (2019). Autophagy suppresses radiation damage by activating PARP-1 and attenuating reactive oxygen species in hepatoma cells. Int. J. Radiat. Biol..

[28] Meng Y.Y., Wu C.W., Yu B., Li H., Chen M., Qi G.X. (2018). PARP-1 Involvement in Autophagy and Their Roles in Apoptosis of Vascular Smooth Muscle Cells under Oxidative Stress. Folia Biol. (Praha).

[29] Son Y.O., Wang X., Hitron J.A., Zhang Z., Cheng S., Budhraja A., Ding S., Lee J.C., Shi X. (2011). Cadmium induces autophagy through ROS-dependent activation of the LKB1-AMPK signaling in skin epidermal cells. Toxicol. Appl. Pharmacol..

[30] Hegedűs C., Boros G., Fidrus E., Kis G.N., Antal M., Juhász T., Janka E.A., Jankó L., Paragh G., Emri G. (2019). PARP1 Inhibition Augments UVB-Mediated Mitochondrial Changes–Implications for UV-Induced DNA Repair and Photocarcinogenesis. Cancers.

[31] Bai P., Canto C., Brunyanszki A., Huber A., Szanto M., Cen Y., Yamamoto H., Houten S.M., Kiss B., Oudart H. (2011). PARP-2 regulates SIRT1 expression and whole-body energy expenditure. Cell Metab..

[32] Bai P., Houten S.M., Huber A., Schreiber V., Watanabe M., Kiss B., de Murcia G., Auwerx J., Menissier-de Murcia J. (2007). Poly(ADP-Ribose) polymerase-2 controls adipocyte differentiation and adipose tissue function through the regulation of the activity of the retinoid X receptor/peroxisome proliferator-activated receptor-gamma heterodimer. J. Biol. Chem..

[33] Rueden C.T., Schindelin J., Hiner M.C., DeZonia B.E., Walter A.E., Arena E.T., Eliceiri K.W. (2017). ImageJ2: ImageJ for the next generation of scientific image data. BMC Bioinform..

[34] Nagy L., Marton J., Vida A., Kis G., Bokor E., Kun S., Gonczi M., Docsa T., Toth A., Antal M. (2018). Glycogen phosphorylase inhibition improves beta cell function. Br. J. Pharmacol..

[35] Ng F., Tang B.L. (2013). Sirtuins’ modulation of autophagy. J. Cell Physiol..

[36] Lee I.H., Cao L., Mostoslavsky R., Lombard D.B., Liu J., Bruns N.E., Tsokos M., Alt F.W., Finkel T. (2008). A role for the NAD-dependent deacetylase Sirt1 in the regulation of autophagy. Proc. Natl. Acad. Sci. USA.

[37] Mohamed J.S., Hajira A., Pardo P.S., Boriek A.M. (2014). MicroRNA-149 inhibits PARP-2 and promotes mitochondrial biogenesis via SIRT-1/PGC-1alpha network in skeletal muscle. Diabetes.

[38] Lagouge M., Argmann C., Gerhart-Hines Z., Meziane H., Lerin C., Daussin F., Messadeq N., Milne J., Lambert P., Elliott P. (2006). Resveratrol Improves Mitochondrial Function and Protects against Metabolic Disease by Activating SIRT1 and PGC-1alpha. Cell.

[39] Kauppinen T.M., Gan L., Swanson R.A. (2013). Poly(ADP-Ribose) polymerase-1 -induced NAD depletion promotes Nuclear Factor-kappaB transcriptional activity by preventing p65 de-acetylation. Biochim. Biophys. Acta.

[40] Menissier-de Murcia J., Ricoul M., Tartier L., Niedergang C., Huber A., Dantzer F., Schreiber V., Ame J.C., Dierich A., LeMeur M. (2003). Functional interaction between PARP-1 and PARP-2 in chromosome stability and embryonic development in mouse. EMBO J..

[41] Bai P., Canto C., Oudart H., Brunyanszki A., Cen Y., Thomas C., Yamamoto H., Huber A., Kiss B., Houtkooper R.H. (2011). PARP-1 Inhibition Increases Mitochondrial Metabolism through SIRT1 Activation. Cell Metab..

[42] Wahlberg E., Karlberg T., Kouznetsova E., Markova N., Macchiarulo A., Thorsell A.G., Pol E., Frostell A., Ekblad T., Oncu D. (2012). Family-wide chemical profiling and structural analysis of PARP and tankyrase inhibitors. Nat. Biotechnol..

[43] Oliver A.W., Ame J.C., Roe S.M., Good V., de Murcia G., Pearl L.H. (2004). Crystal structure of the catalytic fragment of murine Poly(ADP-Ribose) polymerase-2. Nucleic Acids Res..

[44] Szántó M., Brunyánszki A., Márton J., Vámosi G., Nagy L., Fodor T., Kiss B., Virag L., Gergely P., Bai P. (2014). Deletion of PARP-2 induces hepatic cholesterol accumulation and decrease in HDL levels. Biochem. Biophys. Acta Mol. Basis Dis..

[45] Roper S.J., Chrysanthou S., Senner C.E., Sienerth A., Gnan S., Murray A., Masutani M., Latos P., Hemberger M. (2014). ADP-ribosyltransferases Parp1 and Parp7 safeguard pluripotency of ES cells. Nucleic Acids Res..

[46] Farres J., Martin-Caballero J., Martinez C., Lozano J.J., Llacuna L., Ampurdanes C., Ruiz-Herguido C., Dantzer F., Schreiber V., Villunger A. (2013). PARP-2 is required to maintain hematopoiesis following sublethal gamma-irradiation in mice. Blood.

[47] Farres J., Llacuna L., Martin-Caballero J., Martinez C., Lozano J.J., Ampurdanes C., Lopez-Contreras A.J., Florensa L., Navarro J., Ottina E. (2015). PARP-2 sustains erythropoiesis in mice by limiting replicative stress in erythroid progenitors. Cell Death Differ..

[48] Yelamos J., Monreal Y., Saenz L., Aguado E., Schreiber V., Mota R., Fuente T., Minguela A., Parrilla P., de Murcia G. (2006). PARP-2 deficiency affects the survival of CD4+CD8+ double-positive thymocytes. EMBO J..

[49] Vida A., Abdul-Rahman O., Miko E., Brunyanszki A., Bai P. (2016). Poly(ADP-Ribose) Polymerases in Aging-Friend or Foe?. Curr. Protein Pept. Sci..

[50] Nozaki T., Fujimori H., Wang J., Suzuki H., Imai H., Watanabe M., Ohura K., Masutani M. (2013). Parp-1 deficiency in ES cells promotes invasive and metastatic lesions accompanying induction of trophoblast giant cells during tumorigenesis in uterine environment. Pathol. Int..

[51] Luo X., Ryu K.W., Kim D.S., Nandu T., Medina C.J., Gupte R., Gibson B.A., Soccio R.E., Yu Y., Gupta R.K. (2017). PARP-1 Controls the Adipogenic Transcriptional Program by PARylating C/EBPbeta and Modulating Its Transcriptional Activity. Mol. Cell.

[52] Zhang T., Berrocal J.G., Yao J., DuMond M.E., Krishnakumar R., Ruhl D.D., Ryu K.W., Gamble M.J., Kraus W.L. (2012). Regulation of Poly(ADP-Ribose) polymerase-1-dependent gene expression through promoter-directed recruitment of a nuclear NAD+ synthase. J. Biol. Chem..

[53] Hu B., Wu Z., Hergert P., Henke C.A., Bitterman P.B., Phan S.H. (2013). Regulation of myofibroblast differentiation by Poly(ADP-Ribose) polymerase 1. Am. J. Pathol..

[54] Butler A.J., Ordahl C.P. (1999). Poly(ADP-Ribose) polymerase binds with transcription enhancer factor 1 to MCAT1 elements to regulate muscle-specific transcription. Mol. Cell Biol..

[55] Vyas D.R., McCarthy J.J., Tsika G.L., Tsika R.W. (2001). Multiprotein complex formation at the beta myosin heavy chain distal muscle CAT element correlates with slow muscle expression but not mechanical overload responsiveness. J. Biol. Chem..

[56] Chacon-Cabrera A., Fermoselle C., Salmela I., Yelamos J., Barreiro E. (2015). MicroRNA expression and protein acetylation pattern in respiratory and limb muscles of Parp-1(−/−) and Parp-2(−/−) mice with lung cancer cachexia. Biochim. Biophys. Acta.

[57] Liang J., Zeng Z., Zhang Y., Chen N. (2019). Regulatory role of exercise-induced autophagy for sarcopenia. Exp. Gerontol..

[58] Margeta M. (2019). Autophagy Defects in Skeletal Myopathies. Annu. Rev. Pathol..

[59] Yelamos J., Schreiber V., Dantzer F. (2008). Toward specific functions of Poly(ADP-Ribose) polymerase-2. Trends Mol. Med..

[60] Kutuzov M.M., Khodyreva S.N., Ilina E.S., Sukhanova M.V., Ame J.C., Lavrik O.I. (2015). Interaction of PARP-2 with AP site containing DNA. Biochimie.

[61] Sukhanova M.V., Abrakhi S., Joshi V., Pastre D., Kutuzov M.M., Anarbaev R.O., Curmi P.A., Hamon L., Lavrik O.I. (2016). Single molecule detection of PARP1 and PARP2 interaction with DNA strand breaks and their poly(ADP-ribosyl) ation using high-resolution AFM imaging. Nucleic Acids Res..

[62] Haenni S.S., Hassa P.O., Altmeyer M., Fey M., Imhof R., Hottiger M.O. (2008). Identification of lysines 36 and 37 of PARP-2 as targets for acetylation and auto-ADP-ribosylation. Int. J. Biochem. Cell Biol..

[63] Marton J., Peter M., Balogh G., Bodi B., Vida A., Szanto M., Bojcsuk D., Janko L., Bhattoa H.P., Gombos I. (2018). Poly(ADP-Ribose) polymerase-2 is a lipid-modulated modulator of muscular lipid homeostasis. Biochim. Biophys. Acta.

[64] Zhang L., Zou J., Chai E., Qi Y., Zhang Y. (2014). Alpha-lipoic acid attenuates cardiac hypertrophy via downregulation of PARP-2 and subsequent activation of SIRT-1. Eur. J. Pharmacol..

[65] Zheng G.D., Hu P.J., Chao Y.X., Zhou Y., Yang X.J., Chen B.Z., Yu X.Y., Cai Y. (2019). Nobiletin induces growth inhibition and apoptosis in human nasopharyngeal carcinoma C666-1 cells through regulating PARP-2/SIRT1/AMPK signaling pathway. Food Sci. Nutr..

[66] Sun Q., Gatie M.I.I., Kelly G.M. (2019). Serum-dependent and independent regulation of PARP2. Biochem. Cell Biol..

[67] Bai P., Canto C. (2012). The role of PARP-1 and PARP-2 enzymes in metabolic regulation and disease. Cell Metab..

[68] Vida A., Marton J., Miko E., Bai P. (2017). Metabolic roles of Poly(ADP-Ribose) polymerases. Semin. Cell Dev. Biol..

[69] Zhou J., Ng S., Huang Q., Wu Y.T., Li Z., Yao S.Q., Shen H.M. (2013). AMPK mediates a pro-survival autophagy downstream of PARP-1 activation in response to DNA alkylating agents. FEBS Lett..

[70] Chen Z.T., Zhao W., Qu S., Li L., Lu X.D., Su F., Liang Z.G., Guo S.Y., Zhu X.D. (2015). PARP-1 promotes autophagy via the AMPK/mTOR pathway in CNE-2 human nasopharyngeal carcinoma cells following ionizing radiation, while inhibition of autophagy contributes to the radiation sensitization of CNE-2 cells. Mol. Med. Rep..

[71] Ethier C., Tardif M., Arul L., Poirier G.G. (2012). PARP-1 Modulation of mTOR Signaling in Response to a DNA Alkylating Agent. PLoS ONE.

[72] Lee H.R., Gupta M.K., Kim D.H., Hwang J.H., Kwon B., Lee H.T. (2016). Poly(ADP-ribosyl) ation is involved in pro-survival autophagy in porcine blastocysts. Mol. Reprod. Dev..

[73] Mateu-Jimenez M., Cucarull-Martinez B., Yelamos J., Barreiro E. (2016). Reduced tumor burden through increased oxidative stress in lung adenocarcinoma cells of PARP-1 and PARP-2 knockout mice. Biochimie.

[74] Arun B., Akar U., Gutierrez-Barrera A.M., N G. (2015). Hortobagyi, and B. Ozpolat. The PARP inhibitor AZD2281 (Olaparib) induces autophagy/mitophagy in BRCA1 and BRCA2 mutant breast cancer cells. Int. J. Oncol..

[75] Cantó C., Sauve A., Bai P. (2013). Crosstalk between Poly(ADP-Ribose) polymerase and sirtuin enzymes. Mol. Asp. Med..

[76] Hwang J.W., Chung S., Sundar I.K., Yao H., Arunachalam G., McBurney M.W., Rahman I. (2010). Cigarette smoke-induced autophagy is regulated by SIRT1-PARP-1-dependent mechanism: Implication in pathogenesis of COPD. Arch. Biochem. Biophys..

[77] Shin B.H., Shin B.H., Lim Y., Oh H.J., Park S.M., Lee S.K., Ahnn J., Kim D.H., Song W.K., Kwak T.H. (2013). Pharmacological activation of Sirt1 ameliorates polyglutamine-induced toxicity through the regulation of autophagy. PLoS ONE.

[78] Hardie D.G. (2011). AMP-activated protein kinase: An energy sensor that regulates all aspects of cell function. Genes Dev..

[79] Lampada A., O’Prey J., Szabadkai G., Ryan K.M., Hochhauser D., Salomoni P. (2017). mTORC1-independent autophagy regulates receptor tyrosine kinase phosphorylation in colorectal cancer cells via an mTORC2-mediated mechanism. Cell Death Differ..

[80] Bernard M., Dieude M., Yang B., Hamelin K., Underwood K., Hebert M.J. (2014). Autophagy fosters myofibroblast differentiation through MTORC2 activation and downstream upregulation of CTGF. Autophagy.

[81] Arias E., Koga H., Diaz A., Mocholi E., Patel B., Cuervo A.M. (2015). Lysosomal mTORC2/PHLPP1/Akt Regulate Chaperone-Mediated Autophagy. Mol. Cell.

[82] Dhar S.K., Bakthavatchalu V., Dhar B., Chen J., Tadahide I., Zhu H., Gao T., Clair D.K.S. (2018). DNA polymerase gamma (Polgamma) deficiency triggers a selective mTORC2 prosurvival autophagy response via mitochondria-mediated ROS signaling. Oncogene.

[83] Canto C., Houtkooper R.H., Pirinen E., Youn D.Y., Oosterveer M.H., Cen Y., Fernandez-Marcos P.J., Yamamoto H., Andreux P.A., Cettour-Rose P. (2012). The NAD(+) Precursor Nicotinamide Riboside Enhances Oxidative Metabolism and Protects against High-Fat Diet-Induced Obesity. Cell Metab..

[84] Vannini N., Campos V., Girotra M., Trachsel V., Rojas-Sutterlin S., Tratwal J., Ragusa S., Stefanidis E., Ryu D., Rainer P.Y. (2019). The NAD-Booster Nicotinamide Riboside Potently Stimulates Hematopoiesis through Increased Mitochondrial Clearance. Cell Stem Cell..

[85] Hipkiss A.R. (2009). NAD+ availability and proteotoxicity. Neuromol. Med..

[86] Huber A., Bai P., Menissier-de Murcia J., de Murcia G. (2004). PARP-1, PARP-2 and ATM in the DNA damage response: Functional synergy in mouse development. DNA Repair.

[87] Vaziri H., Dessain S.K., Eaton E.N., Imai S.I., Frye R.A., Pandita T.K., Guarente L., Weinberg R.A. (2001). hSIR2 (SIRT1) functions as an NAD-dependent p53 deacetylase. Cell.

[88] Li X., Klaus J.A., Zhang J., Xu Z., Kibler K.K., Andrabi S.A., Rao K., Yang Z.J., Dawson T.M., Dawson V.L. (2010). Contributions of Poly(ADP-Ribose) polymerase-1 and -2 to nuclear translocation of apoptosis-inducing factor and injury from focal cerebral ischemia. J. Neurochem..

[89] Kofler J., Otsuka T., Zhang Z., Noppens R., Grafe M.R., Koh D.W., Dawson V.L., Menisser-de Murcia J., Hurn P.D., Traystman R.J. (2006). Differential effect of PARP-2 deletion on brain injury after focal and global cerebral ischemia. J. Cereb. Blood Flow Metab..

[90] Czarny P., Pawlowska E., Bialkowska-Warzecha J., Kaarniranta K., Blasiak J. (2015). Autophagy in DNA damage response. Int. J. Mol. Sci..

[91] Gueguen Y., Bontemps A., Ebrahimian T.G. (2019). Adaptive responses to low doses of radiation or chemicals: Their cellular and molecular mechanisms. Cell Mol. Life Sci..

